# Effectiveness of genomic prediction on milk flow traits in dairy cattle

**DOI:** 10.1186/1297-9686-44-24

**Published:** 2012-07-30

**Authors:** Kent A Gray, Joseph P Cassady, Yijian Huang, Christian Maltecca

**Affiliations:** 1 North Carolina State University, Campus Box 7621, Raleigh, NC 27695, USA

## Abstract

**Background:**

Milkability, primarily evaluated by measurements of milking speed and time, has an economic impact in milk production of dairy cattle. Recently the Italian Brown Swiss Breeders Association has included milking speed in genetic evaluations. The main objective of this study was to investigate the possibility of implementing genomic selection for milk flow traits in the Italian Brown Swiss population and thereby evaluate the potential of genomic selection for novel traits in medium-sized populations. Predicted breeding values and reliabilities based on genomic information were compared with those obtained from traditional breeding values.

**Methods:**

Milk flow measures for total milking time, ascending time, time of plateau, descending time, average milk flow and maximum milk flow were collected on 37 213 Italian Brown Swiss cows. Breeding values for genotyped sires (n = 1351) were obtained from standard BLUP and genome-enhanced breeding value techniques utilizing two-stage and single-step methods. Reliabilities from a validation dataset were estimated and used to compare accuracies obtained from parental averages with genome-enhanced predictions.

**Results:**

Genome-enhanced breeding values evaluated using two-stage methods had similar reliabilities with values ranging from 0.34 to 0.49 for the different traits. Across two-stage methods, the average increase in reliability from parental average was approximately 0.17 for all traits, with the exception of descending time, for which reliability increased to 0.11. Combining genomic and pedigree information in a single-step produced the largest increases in reliability over parent averages: 0.20, 0.24, 0.21, 0.14, 0.20 and 0.21 for total milking time, ascending time, time of plateau, descending time, average milk flow and maximum milk flow, respectively.

**Conclusions:**

Using genomic models increased the accuracy of prediction compared to traditional BLUP methods. Our results show that, among the methods used to predict genome-enhanced breeding values, the single-step method was the most successful at increasing the reliability for most traits. The single-step method takes advantage of all the data available, including phenotypes from non-genotyped animals, and can easily be incorporated into current breeding evaluations.

## Background

The inclusion of genomic information in models for prediction of genetic merit is expected to result in increased accuracies of prediction. In 2001, Meuwissen et al. [1] described how breeding values can be predicted from marker data alone in order to obtain what are now commonly known as direct genomic values (DGV). These values are calculated as the sum of the effects of dense genetic markers across the genome, capturing all quantitative trait loci (QTL) that contribute to variation in the trait. Implementation of DGV in genetic evaluations and selection indices is commonly referred to as genomic selection.

Today, genomic selection programs are routinely implemented in the United States, Canada, New Zealand, France, Netherlands, Denmark, Norway and Sweden. Wiggans et al. [2] reported an increase in reliabilities of US genomic predictions using an Illumina 50 k panel [3] as compared to predictions of genetic merit based on traditional parental averages.

Milkability belongs to the group of traits termed functional traits, which also include health, feed efficiency, fertility, and calving ease. Milkability is defined as the ease of milking of dairy cows and is usually measured as milking speed, either recorded objectively or through subjective measures [4,5]. Milking speed or flows measured using electronic measuring devices are not collected on a frequent basis and can be considered novel traits. Other indicators of milkability include flow measures such as Average Milk Flow (AVGF) and Maximum Milk Flow (MMF) [6].

Milkability measures, either as milking flow or speed, have long been recognized as relevant criteria in animal selection [7,8], due to their impact on management costs of milking cows [9]. An increase in MMF or AVGF, or a reduction in total milking time (TMT), results in a reduction of milking labor requirements and in an increase in the efficiency of automatic milking systems [10]. Considering that labor accounts for a large fraction of the total costs of milk production, it is not a surprise that Prins et al. [11] estimated economic values for milking time to range from 1.63 to 25.97€ /minute/cow/year, depending on the size of the milking parlor.

Milking speed and milking time significantly influence the farmer’s economic bottom line and can be improved through selection [7,8]. Indeed, in the past 50 years, milk flow has been actively selected for in some dairy populations [12]. However, due to the cost and labor associated with collecting milk flow data, it has been difficult to make substantial progress compared to other traits.

To our knowledge, the use of molecular information to select for milkability based on milk flow and milking time data has not been investigated. Thus, the main objective of our study was to evaluate the potential of genomic selection (GS) for this relatively novel trait in a medium-sized population, i.e. milk flow collected in Italian Brown Swiss cattle. First, breeding values for milkability traits were predicted using single nucleotide polymorphism (SNP) marker genotypes; second, the reliability of these breeding values was evaluated for sires with a relatively small number of daughters with phenotypic information; third, the reliabilities of alternative methods to predict breeding values were compared to EBV obtained from traditional BLUP (Best Linear Unbiased Prediction) methods and finally, differences between methods were evaluated based on the reliabilities of predictions obtained.

## Methods

### Data

**Figure 1:**
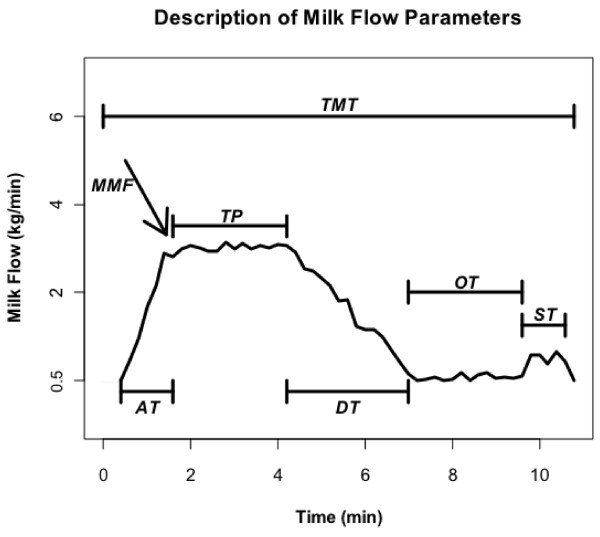
**Description of milk flow parameters. **TMT: Total Milking Time; AT: Ascending Time; TP: Time of Plateau; DT: Descending Time; MMF: Maximum Milk Flow; AVGF: Average Milk Flow.

Data for this study were provided by the Italian Brown Swiss Breeders Association and included information spanning 13 years between 1997 and 2009. The dataset included 37 213 cows, daughters of 2361 sires and 30 231 dams with pedigree information spanning seven generations. Milk flow was measured once for each cow using a portable milk flow recorder (LactoCorder, WMB AG, Balgach, Switzerland). Milk flow characteristics were detected every 0.7 s and saved at intervals of 2.8 s. To describe the overall pattern of milk removal, milk flow was divided into six phases(Figure 1): 1) ascending time (AT), period of time from when milk flow reaches a value greater than 0.5 kg/min until it plateaus; 2) time of plateau (TP), period of time with a steady milk flow; 3) descending time (DT), period of time for the milk flow at the end of TP to reach a value below 0.2 kg/min; 4) over-milking time (OT), period of time necessary for the milking machine to be detached from the udder, once the milk flow is below 0.2 kg/min; 5) stripping time, period at end of milking, with milk flow greater than 0.2 kg/min and lasting for at least 4.2 s; and 6) over-milking time after stripping, period after stripping during which the milk flow decreases to below 0.2 kg/min and the milking machine is removed. In addition, maximum milk flow (MMF) was recorded as the maximum flow preceding TP. Thus the six traits investigated in this study were: TMT, AT, TP, DT, MMF and average milk flow (AVGF). A complete description of data collection and data editing for these traits is reported in Gray et al. [5].

A total of 1351 bulls that were genotyped in the Italian Brown Swiss population had direct relationship ties with cows that had milk flow data. Bulls with daughters in the dataset had an average of 28 ± 2.6 daughters with milk flow data available for analysis. Bulls were genotyped using the Illumina Bovine SNP50BeadChip [13]. After quality checking, 34 052 SNP spanning 29 bovine autosomes remained for the analysis. Markers with a call rate < 0.90, markers with a minor allele frequency (MAF) < 0.05, and markers violating Hardy Weinberg equilibrium test (Chi-square > 300 with 1 d.f.) were discarded from the analysis. The average number of SNP per chromosome was 1175.

### Statistical analyses

#### Two-stage genomic selection

Genomic predictions were first computed using a multi-step approach [14]. First, a traditional evaluation of milking traits was performed. Using a six-trait animal model similar to the model described in Gray et al. [5], traditional breeding values and parent averages for the genotyped bulls were predicted for TMT, AT, TP, DT, MMF and AVGF applying the BLUP methodology with ASREML [15]. Genotyped animals were then split into a training and a validation dataset depending on the reliability of their BLUP EBV. Genotyped sires with a reliability higher than 0.50 for TP and 0.60 for the remaining traits were included in the training dataset, which amounted to separate older sires from young sires with less progeny, i.e. older sires were assigned to the training dataset and the remaining younger animals to the validation dataset.

Pseudo-phenotypes (dEBV) were obtained as deregressed EBV free of the effects of parent average and adjusted for the number of daughters contributing to the EBV for the data vector used in genomic predictions [16].

In the second step, the dEBV were then analyzed for prediction of genomic EBV including a genomic relationship matrix in place of the traditional numerator relationship matrix in the mixed model equations [14], which will be referred to as GBLUP.

The genomic relationship matrix **G** was constructed using the formula $G=\frac{ZZ^{\prime }}{2\Sigma p_{i}(1-p_{i})}$, where *p*_*i*_ is the frequency of marker *i*, estimated from all genotyped sires, and **Z** is the matrix of marker codes (0/1/2) adjusted by setting the mean for each SNP across genotypes to 0 by subtracting **P** defined as a matrix with allele frequencies expressed as a difference from 0.5 and multiplied by 2, such that column *i* of **P** is $2(p_{i}-0.5)$[16]. With GBLUP, predicted breeding values were obtained for animals in the validation dataset through **G**^-1^. Using ASREML software [15], records from the training population entered the mixed model equations as the **y** vector, solving for the predicted breeding values.

Two different non-linear prediction approaches, Bayes-A, and Bayesian LASSO (Least Absolute Shrinkage and Selection Operator), were used to estimate a genetic variance component for each marker, accounting for a non-normal prior distribution. A comparison of these two methods is in Cleveland et al. [17].

The general structure of the models in matrix form was:

y=1μ+Xβ+e

**y**: vector of de-regressed breeding values for TMT, AT, TP, DT, MMF and AVGF; *μ*: overall mean; *β*: vector of additive effects for each marker; **X**: matrix of genotypes coded as number of copies of an arbitrary allele (0, 1, and 2) for each SNP; **e**: vector of residuals assumed normal with variance weighted as outlined by Garrick et al. [16] with a *c* constant for the genetic variance unaccounted for by the markers set at 0.4 after an exploratory analysis (data not shown).

A flat (1) prior was assigned to *μ*, while the prior distribution for $\sigma_{e}^{2}$ was assumed inverted chi-square with 4 degrees of freedom and an expectation equal to the value used in the traditional BLUP evaluation with individual cow records.

The remaining prior structure was:

$$\beta_{i}:N(0,\sigma_{g_{i}}^{2})$$

for the i^th^ SNP,

$$\sigma_{g_{i}}^{2}\sim \text{inv}-\chi^{2}(\sigma_{g_{i}}^{2}|\nu /s^{2})$$

for the BayesA approach and

$$\sigma_{g_{i}}^{2}\sim \text{Exp}(\sigma_{g_{i}}^{2}|2/\lambda_{i}{}^{2})$$

for the LASSO approach.

In the current analysis, a straightforward generalization of the BayesA method was applied, in which scale parameter *s*^2^ and degrees of freedom *ν* were treated as unknown and were estimated from the data [18]. They were assigned a uniform prior in the interval (0,1] for *ν* and a uniform prior for *s* for the range of (0,Q], with Q being 100. At each round of the Gibbs sampler that was implemented, samples of *s*^*2*^ where obtained from $\text{Gamma}(s^{2}|\sigma_{g_{i}}^{2},\nu )$*.* Since the *ν* parameter does not have a closed form, parameter samples were obtained at each round of the sampler with a Metropolis step

$$(\nu |y,\mu ,\beta ,\sigma_{g_{i}}^{2},\sigma_{e}^{2})\text{.}$$

The pseudo-code and a summary of posterior results for scale and degrees of freedom for this step are provided as additional data [see Additional file 1].

The λ parameter in the LASSO approach was assigned a gamma prior distribution *Gamma (*0.05,1.0*)*, so the prior of λ was essentially uniform over a wide range of values [19]. A Gibbs sampling algorithm was implemented in R to obtain samples from the joint posterior distribution [20].

Marker effect estimates were obtained using the above models within the training population and were then applied to the validation dataset to predict genomic breeding values. Assuming a completely additive model, marker effects were summed across the entire genome for each animal to obtain the DGV. Genome-enhanced breeding values (GEBV) were obtained by combining DGV and parental averages, as outlined by Saatchi et al. [21].

#### Single-step approach

As an alternative to the two-step approach, Misztal et al. [22] proposed a unified single-step approach, which automatically combines genomic and phenotypic information into a single set of equations. This approach is basically a modification of current animal model genetic evaluation methodology, in which the inverse of the relationship matrix **A**^-1^ is modified. The resulting matrix, referred to as **H**^-1^, is obtained by simply adding the difference between the inverse of the genomic relationship matrix and the inverse of the pedigree-based relationship matrix for genotyped animals to the corresponding block in the inverse of pedigree-based relationship among all animals [23]:

$$H^{-1}=A^{-1}+\left[\begin{array}{cc} 0 & 0\\ 0 & G^{-1}-A_{22}{}^{-1} \end{array}\right]$$

where $A_{22}^{-1}$ is the inverse of the pedigree-based relationship matrix of genotyped animals. Using ASREML software [15], EBV were obtained by substituting the inverse numerator relationship matrix (**A**^-1^**)** with a user defined matrix (**H**^-1^) in a 6-trait multivariate model. In this study, **G**^-1^ was used to compute **H**^-1^ and breeding values predicted from this method will be referred to as **HBLUP**. Scaling factors have been used to control potential bias in the **H**^-1^[23,24]. In our analysis, scaling factors equal to 0.5, 0.7 and 0.9 were employed, as suggested by Forni et al. [24]. Scaling did not result in any overall change and only the results from using a scaling factor equal to 0.9 will be presented.

Reliabilities (r^2^) for dEBV predicted from BLUP, were computed as $1-\frac{SEP^{2}}{(1+f_{i})\sigma_{a}^{2}}$ where *SEP* = standard error of the prediction, *f*_*i*_ = inbreeding coefficient for animal *i* and $\sigma_{a}^{2}$ = genetic variance [15]. Reliabilities for the genomic predictions were measured from accuracies calculated according to Saatchi et al. [21] as: $r^{2}{}_{\mathrm{PG}}={\left(\frac{{\hat{\sigma }}_{\text{dEBV},\text{GBV}}}{\sqrt{\sigma^{2}{}_{a}{\hat{\sigma }}^{2}{}_{\mathrm{GEBV}}}}\right)}^{2}$where $\sigma^{2}{}_{a}$ is the genetic variance as obtained with the complete data, ${\hat{\sigma }}_{\text{dEBV},\text{GEBV}}$ is the covariance between GEBV and deregressed EBV and ${\hat{\sigma }}^{2}{}_{\mathrm{GEBV}}$ is the GEBV variance.

#### Comparison of dEBV models with genome-enhanced models

Pearson correlations were estimated between dEBV from validation individuals with GEBV obtained from the other models to measure the relationship between EBV obtained from different models. Differences in model performance for the dEBV calculated with GBLUP, Bayesian LASSO, BayesA and HBLUP were evaluated by regressing dEBV of validation individuals on GEBV obtained from the other models.

## Results and discussion

Estimates of heritability and genetic correlations from the six-trait pedigree-based animal model are reported in Table 1. Estimates were low for the milking time traits, except for TP and milk flow traits, which had moderate heritabilities. Estimated heritabilities were in agreement with other studies [6,25].

**Table 1:** Heritabilities (on diagonal), genetic (above diagonal) and phenotypic correlations (below diagonal)

	**MMF**	**AVGF**	**AT**	**TP**	**DT**	**TMT**
MMF	**0.11**	0.95	−0.61	0.85	0.73	−0.88
AVGF	0.83	**0.27**	−0.58	−0.84	−0.58	−0.90
AT	−0.01	−0.03	**0.02**	−0.76	−0.26	0.82
TP	0.40	−0.27	−0.26	**0.32**	0.31	−0.93
DT	0.39	−0.36	−0.04	−0.15	**0.05**	−0.44
TMT	−0.34	−0.32	0.17	−0.59	−0.10	**0.42**

MMF: Maximum Milk Flow; AVGF: Average Milk Flow; AT: Ascending Time; TP: Time at Plateau; DT: Descending Time; TMT: Total Milking Time.

### Comparison of reliabilities

All comparisons were based on reliabilities of EBV and parental averages estimated from the validation set. Parental averages in the data employed here include information corresponding to genotyped bulls. In order to eliminate it from the cumulative information on the parents, adjusted parent averages (aPA) were obtained following what proposed by Garrick et al. [16].

Breeding values predicted from marker data had a better predictive ability than estimates of aPA from pedigree-based relationships. The average increase of GEBV reliabilities ranged from 0.11 to 0.18 over aPA when using GBLUP and from 0.12 to 0.2 when using BayesA and Bayesian LASSO (Table 2).

**Table 2:** Reliability of estimated breeding values for sires in the validation set

**Trait**	**aPA**	**dEBV**^**3**^	**GBLUP**	**BayesA**	**Bayesian LASSO**	**HBLUP**
TMT	0.29	0.43	0.44	0.47	0.48	0.49
AT	0.26	0.44	0.40	0.43	0.44	0.50
TP	0.30	0.47	0.43	0.47	0.49	0.51
DT	0.23	0.34	0.34	0.35	0.35	0.37
MMF	0.30	0.42	0.48	0.47	0.48	0.50
AVGF	0.29	0.43	0.46	0.48	0.49	0.50

TMT: Total Milking Time; AT: Ascending Time; TP: Time of Plateau; DT: Descending Time; MMF: Maximum Milk Flow; AVGF: Average Milk Flow; dEBV: de-regressed breeding values; GBLUP: Genomic Breeding Value; Bayesian LASSO: Bayesian Least Absolute Shrinkage and Selection Operator; HBLUP: single-step combined phenotypic and genotypic information.

Among the six traits analyzed, the increase in reliability was largest for MMF and AVGF when GEBV were calculated with GBLUP. MMF and AVGF also had the largest heritabilities compared to AT, TP, DT and TMT.

BayesA and Bayesian LASSO methods produced near identical mean reliabilities for all milk flow traits, i.e. with Bayesian LASSO: 0.44 for AT, 0.49 for TP, 0.35 for DT, 0.48 for MMF, 0.49 for AVGF and 0.48 for TMT, while with BayesA they decreased slightly by 0.01 for AT and AVGF and by 0.02 for TP (Table 2).

With the single-step approach, reliabilities were either identical or increased slightly compared to the other prediction methods for all traits. Increases in reliability ranged from 0.14 to 0.24 over aPA reliabilities and from 0.01 to 0.07 over reliabilities from non-linear methods (Table 2). It should be noted that all two-step approaches were univariate models, while the one-step approach was a multi-trait model. Most implementations of two-step genomic selection methods are currently based on single-trait analyses but multivariate approaches are emerging. In this work, we did not consider a multivariate approach for the two-step methods although it would be interesting to include this option. Therefore, increases in reliability are probably partly due to the modeling of covariances among traits in the analysis. Furthermore, the slight increase in reliability observed here could also be attributed to the fact that measured phenotypes of cows were used instead of pseudo-phenotypes derived from breeding values of the sires. When pseudo-phenotypes are derived from animals with small progeny numbers and are implemented in evaluations involving the two-step method, EBV tend to be less accurate.

### Comparison of breeding values

Simple linear models were used to regress dEBV on genomic breeding values obtained from GBLUP, HBLUP, Bayesian LASSO and BayesA models (Table 3). Correlations of breeding values obtained from PBLUP and GBLUP predictions ranged across traits from 0.70 to 0.86, from 0.70 to 0.86 between PBLUP and both Bayesian LASSO and BayesA and from 0.87 to 0.93 between PBLUP and HBLUP (Table 3).

**Table 3:** Comparison of estimated breeding values obtained using PBLUP with estimates from GBLUP, HBLUP, Bayesian LASSO and BayesA

**Two-stage methods**						
GBLUP						
	**TMT**	**AT**	**TP**	**DT**	**MMF**	**AVGF**
Slope	0.88	0.879	0.850	0.850	0.865	0.812
Correlation	0.780	0.861	0.800	0.720	0.770	0.702
Bayesian LASSO						
Slope	0.870	0.922	0.800	0.810	0.860	0.862
Correlation	0.780	0.814	0.830	0.770	0.730	0.717
BayesA						
Slope	0.870	0.892	0.840	0.824	0.881	0.901
Correlation	0.770	0.863	0.780	0.740	0.780	0.703
**Single-step method**						
HBLUP						
Slope	0.842	0.869	0.757	0.873	0.775	0.814
Correlation	0.907	0.929	0.867	0.925	0.880	0.897

Regression of dEBV on predicted genomic breeding values obtained with GBLUP, Bayesian LASSO, BayesA and HBLUP; GBLUP: similar to traditional BLUP predictions with the exception that genomic relationships are utilized instead of pedigree relationships; HBLUP: similar to traditional with the exception that a combined relationship matrix is computed using both pedigree and genomic information; TMT: Total Milking Time; AT: Ascending Time; TP: Time of Plateau; DT: Descending Time; MMF: Maximum Milk Flow; AVGF: Average Flow.

It is likely that an increase in the number of progeny per sire within the validation set would reduce the difference between PBLUP and the two-step methods. It should be noted that in this study, the size of the training dataset was limited compared to studies performed on Holstein cattle. However, even with the limited size of the Italian Brown Swiss population, the breeding values obtained with the HBLUP method showed stronger correlations than other models indicating that its features are very robust and that it can be applied in the case of small populations.

## Conclusions

Breeding values for milk flow traits estimated from genomic markers show an increase in reliability in most cases compared to traditional pedigree-based evaluations. Most of the increase in reliability can be attributed to the improved estimation of Mendelian sampling.

The choice of priors in the analysis of non-linear methods evaluated here, did not significantly affect reliabilities. In most cases 2-step non-linear models slightly outperformed GBLUP. Some advantages associated with using two-step non-linear procedures include flexibility in the incorporation of genomic information by maintaining a separation from traditional breeding value estimation. Although traditional breeding value estimation has been used in numerous populations and diverse situations, it was not the best method in our study. Two-step methods can be useful to continue work in genome-wide association studies and are easily scalable as the number of animals and markers increases. However, these methods tend to be slow due to the necessity to sample often more than 100 k rounds in an MCMC procedure. They are also heavily dependent on parameters specified by assumptions given by the user that could be incorrect and are not easy to implement in more complicated models, which could include maternal effects or random regression.

Our results suggest that, among all the methods evaluated, the single-step method was the most successful at increasing the reliability for all traits. This method takes advantage of all the data available and is easily incorporated into current breeding evaluations. Although milk flow is a fairly novel trait and the measurements used in this study were obtained on a relatively small population compared to other dairy breeds, selection based on single-step methods is expected to provide the best response with the greatest flexibility.

## Competing interests

The authors declare that they have no competing interests.

## Authors’ contributions

KAG performed analysis and drafted the manuscript. YH helped in the analysis and in drafting the manuscript. JPC helped draft and revise the manuscript; CM designed the study helped in the analyses and in drafting the manuscript. All authors read and approved the final version.

## Supplementary Material

Additional file 1 **Pseudo-code and summary of posterior results for scale and degrees of freedom. **The data provided include R pseudo-code for the sampling of degrees of freedom and scale as well as posterior mean and distribution for the two parameters. Click here for file

## References

[B1] Meuwissen TH Hayes BJ Goddard ME Prediction of total genetic value using genome-wide dense marker maps Genetics 2001 157 1819 1829 11290733 10.1093/genetics/157.4.1819 PMC1461589

[B2] Wiggans GR VanRaden PM Cooper TA The genomic evaluation system in the United States: Past, present, future J Dairy Sci 2011 94 3202 3211 10.3168/jds.2010-3866 21605789

[B3] BovineSNP50 Genotyping BeadChip http://www.illumina.com/Documents/products/datasheets/datasheet_bovine_snp5O.pdf

[B4] Meyer K Burnside EB Scope for a subjective assessment of milking speed J Dairy Sci 1987 70 1061 1068 10.3168/jds.S0022-0302(87)80112-1

[B5] Gray KA Vacirca F Bagnato A Samoré AB Rossoni A Maltecca C Genetic evaluations for measures of the milk-flow curve in the Italian Brown Swiss population J Dairy Sci 2011 94 960 970 10.3168/jds.2009-2759 21257064

[B6] Guler O Yanar M Aydin R Bayram B Dogru U Kopuzlu S Genetic and environmental parameters of milkability traits in Holstein Friesian cows J Anim Vet Adv 2009 8 143 147

[B7] Bruckmaier R Rothenanger E Blum J Milking characteristics in dairy cows of different breeds from different farms and during the course of lactation J Anim Breed Genet 1995 112 293 302 10.1111/j.1439-0388.1995.tb00569.x

[B8] Miller RH Pearson RE Weinland BT Fulton LA Genetic parameters of several measures of milk flow-rate and milking time J Dairy Sci 1976 59 957 964 10.3168/jds.S0022-0302(76)84304-4 1270653

[B9] Groen AF Steine T Colleau JJ Pedersen J Pribyl J Reinsch N Economic values in dairy cattle breeding, with special reference to functional traits. Report of an EAAP working group Livest Prod Sci 1997 49 1 21 10.1016/S0301-6226(97)00041-9

[B10] Dondenhoff J Sprengel D Duda J Dempfle L Studies on genetic evaluation of udder health using the Lacto Corder Zuchtungskunde 1999 71 459 472

[B11] Prins D Groen AF Saatkamp H Economic value of milkability in dairy cattle.MSc thesis Wageningen University 2002 Wageningen Institute of Animal Sciences

[B12] Politiek D Observations on the practicality of measuring ease of milking in cows and its variations, also some reflections on the heritability of this factor Proceedings of the 8th International Congress on Animal Production: 1961; Hamburg 1961 148 166

[B13] Matukumalli LK Lawley CT Schnabel RD Taylor JF Allan MF Heaton MP O'Connell J Moore SS Smith TPL Sonstegard TS Van Tassell CP Development and characterization of a high density SNP genotyping assay for cattle PLoS One 2009 4 e5350 10.1371/journal.pone.0005350 19390634 PMC2669730

[B14] VanRaden PM Efficient methods to compute genomic predictions J Dairy Sci 2008 91 4414 4423 10.3168/jds.2007-0980 18946147

[B15] Gilmour AR Gogel BJ Cullis BR Thompson R ASReml User Guide Release 3.0 2009 http://www.vsni.co.uk

[B16] Garrick DJ Taylor JF Fernando RL Deregressing estimated breeding values and weighting information for genomic regression analyses Genet Sel Evol 2009 41 55 10.1186/1297-9686-41-55 20043827 PMC2817680

[B17] Cleveland M Forni S Deeb N Maltecca C Genomic breeding value prediction using three Bayesian methods and application to reduced density marker panels BMC Proc 2010 4 S6 10.1186/1753-6561-4-S1-S6 20380760 PMC2857848

[B18] Yi N Xu S Bayesian LASSO for quantitative trait loci mapping Genetics 2008 179 1045 1055 10.1534/genetics.107.085589 18505874 PMC2429858

[B19] Park T Casella G The Bayesian LASSO J Am Statist Assoc 2008 103 681 686 10.1198/016214508000000337

[B20] R Development Core Team R: A Language and Environment for Statistical Computing 2010 R Foundation for Statistical computing, Vienna

[B21] Saatchi M McClure M McKay S Rolf M Kim J Decker J Taxis T Chapple R Ramey H Northcutt S Bauck S Woodward B Dekkers JCM Fernando RL Schnabel RD Garrick DJ Taylor JF Accuracies of genomic breeding values in American Angus beef cattle using K-means clustering for cross-validation Genet Sel Evol 2011 43 40 10.1186/1297-9686-43-40 22122853 PMC3250932

[B22] Misztal I Legarra A Aguilar I Computing procedures for genetic evaluation including phenotypic, full pedigree, and genomic information J Dairy Sci 2009 92 4648 4655 10.3168/jds.2009-2064 19700728

[B23] Aguilar I Misztal I Johnson DL Legarra A Tsuruta S Lawlor TJ Hot topic: A unified approach to utilize phenotypic, full pedigree, and genomic information for genetic evaluation of Holstein final score J Dairy Sci 2010 93 743 752 10.3168/jds.2009-2730 20105546

[B24] Forni S Aguilar I Misztal I Different genomic relationship matrices for single-step analysis using phenotypic, pedigree and genomic information Genet Sel Evol 2011 43 1 10.1186/1297-9686-43-1 21208445 PMC3022661

[B25] Aydin R Yanar M Guler O Yuksel S Ugur F Turgut L Study on milkability traits in Brown Swiss cows reared in Eastern region of Turkey J Vet Anim Adv 2008 7 1218 1222

