# Supplementary material for: Effectiveness of genomic prediction on milk flow traits in dairy cattle
Source: Genet Sel Evol. 2012 Jul 30;44(1):24. doi: 10.1186/1297-9686-44-24 (PMC3507773; doi:10.1186/1297-9686-44-24)
Supplement: Additional file 1 — Pseudo-code and summary of posterior results for scale and degrees of freedom. The data provided include R pseudo-code for the sampling of degrees of freedom and scale as well as posterior mean and distribution for the two parameters. [file 1297-9686-44-24-S1.doc]

**Supplemental Material**

R pseudo code to update the S parameter

S<-sqrt(rgamma(n=1,shape=(nmarkers*nu/2),rate=(nu/2*sum(1/gvar))))

R pseudo code to update the nu parameter

nu.update<-function(sigma.jump.nu,nu,s,gvar){

nu.inv.star <- rnorm(1, 1/nu, sigma.jump.nu)

if(nu.inv.star<= 0 | nu.inv.star >1){

p.jump <- 0

}

else {

nu.star <- 1/nu.inv.star

log.post.old <- log.post (gvar, s, nu)

log.post.star <- log.post (gvar,s,nu.star)

r <- exp (log.post.star - log.post.old)

nu <- ifelse (runif(1) < r, nu.star, nu)

p.jump <-min(r,1)

}

return (list(nu, p.jump))

}

R pseudo code for the Log posterior density (only the part proportional to nu is calculated )

log.post <- function (gvar,s, nu){

sum(0.5*nu*log(nu/2) + nu*log(s) -lgamma(nu/2) - (nu/2+1)*log(gvar) - (.5*nu*s^2)/(gvar))

}

Note

Nmarkers= # Markers

gvar= Markers variance

nu= df parameter

S= scale parameter

**Table S1: Posterior mean of S values for Bayes-A**.

|  | S |
| --- | --- |
| TMT | 0.0027 |
| AT | 0.0023 |
| TP | 0.0022 |
| DT | 0.0014 |
| MMF | 0.0025 |
| AVGF | 0.0032 |

**Figure S1: Posterior distribution of $\nu$values.**
